# Welcome editorial from the new Editor-in-Chief

**DOI:** 10.1017/qrd.2026.10020

**Published:** 2026-05-06

**Authors:** Carol V. Robinson, Emma Lalande

**Affiliations:** Kavli Institute for Nanoscience Discovery and Department of Chemistry, University of Oxford, UK

There is nothing more exciting than understanding the world around us – whether physical, biological, or both. QRB Discovery is a bastion for disseminating groundbreaking results about the world we live in. It has been since its inception in 2020, when it was instituted as the journal of Biophysical Discovery under Professor Bengt Nordén.

Since then, QRB Discovery has established itself as a publication whose scope extends beyond traditional perspectives, creating a dedicated space for high-quality, speculative research that crosses disciplinary boundaries.

The world in 2026 faces myriad complex problems and grand challenges. We are witnessing an outpacing technological evolution, the eruption of warfare, the resurgence of human diseases, and the degradation of our planet’s health. We must not underestimate the value of discovery science in contributing to understanding, stability, and action in unprecedented times.

For our work to make an impact in these times, we must bring together people with diverse scientific backgrounds, knowledge, and experiences. I’ve seen that the most inspiring and often highest impact discoveries take a level of boldness, grounded in the curiosity of answering fundamental questions. We must use our collective curiosity and creativity to explore bold, new ideas together. Critically, we must share these ideas with each other, collaborating within our local and broader communities.

With those principles in mind, we are committed to reproducible open-access research – positive and negative findings together – and transparent, expert peer review. We will publish disruptive research while upholding the strictest scientific standards.

Our standards will be centered on careful consideration of the potential use cases and wider ethical aspects of the work we produce and the results that we publish. We encourage explicit attention to responsible practice and the societal implications of scientific findings.

Our thematic strategy will showcase emerging fields, discussions, and interdisciplinary perspectives. A personal goal is to support young researchers, particularly where interesting findings are yet to be fully understood, and where interdisciplinary research takes longer to generate a track record. I still remember the thrill of having my first independent paper published when no one had heard of me. It was tremendously exciting for me and my student at that time.

Promoting the voices of first-time authors and underrepresented groups is crucial for the field’s future. How do we achieve this? We will support and encourage young researchers through greater engagement on the editorial board. Additionally, we aim to widen accessibility to our journal content by mentoring first-time submissions and creating editorial policies that facilitate constructive feedback.

In this next editorial term, it is our hope that QRB Discovery will expand as a focal point: at the heart of prescient topics, championing young minds in interdisciplinary research, and serving as a touchstone for the global biophysics community.

